# Supplementary material for: Decorin Promotes Nascent Proteoglycan Retention in Cartilage Matrix by Strengthening Collagen II-Aggrecan Integration
Source: bioRxiv. 2025 Nov 13:2025.11.12.688145. Preprint. [Version 1] doi: 10.1101/2025.11.12.688145 (PMC12642626; doi:10.1101/2025.11.12.688145)
Supplement: Supplement 1 [file media-1.pdf]

Supporting Information

Decorin Promotes Nascent Proteoglycan Retention in Cartilage Matrix by Strengthening Collagen II-Aggrecan Integration

Thomas Li,[1] Gabriela Canziani,[2] Yuchen Liu,[1] Neil Patel,[1] Biao Han,[1] Mingyue Fan,[1] Annie Porter,[3] Bryan Kwok,[1] Chao Wang,[1] Qing Li,[1] David E. Birk,[4] Renato V. Iozzo,[5] Irwin M. Chaiken,[2] X. Lucas Lu,[3] Robert L. Mauck,[6,7] and Lin Han[1,*]

[1]*School of Biomedical Engineering, Science and Health Systems, Drexel University, Philadelphia, PA 19104, United States*

[2]*Department of Biochemistry and Molecular Biology, College of Medicine, Drexel University, Philadelphia, PA 19102, United States*

[3]*Department of Mechanical Engineering, University of Delaware, Newark, DE 19716, United States*

[4]*Department of Molecular Pharmacology and Physiology, Morsani School of Medicine, University of South Florida, Tampa, FL 33612, United States*

[5]*Department of Pathology and Genomic Medicine, Sidney Kimmel Medical College, Thomas Jefferson University, Philadelphia, PA 19107, United States*

[6]*McKay Orthopaedic Research Laboratory, Department of Orthopaedic Surgery, Perelman School of Medicine, University of Pennsylvania, Philadelphia, PA 19104, United States*

[7]*Translational Musculoskeletal Research Center, Corporal Michael J. Crescenz Veterans Administration Medical Center, Philadelphia, PA 19104, United States*

*Correspondence and requests for materials should be addressed to:

Dr. Lin Han

Phone: (215)571-3821

Fax: (215)895-4983

Email: lh535@drexel.edu.

Table S1. Summary of averaged values and statistical analysis outcomes of the mean GAL intensity, I_{mean}, extracted from high-resolution confocal images of click-labeled femoral head cartilage explants of age-matched wild-type (WT) and decorin-null ($Dcn^{-/-}$) mice over 6 days of culture, shown as mean ± 95% CI from values averaged by each explant. The *p*-values were calculated using the linear mixed-effect model, followed by Holm-Bonferroni correction for multiple contrasts between genotypes and treatments, and Tukey-Kramer correction for multiple comparisons across culture time points for each genotype and treatment.

I_{mean} (GAL) (a.u.)	Untreated					
	WT			$Dcn^{-/-}$		
	mean ± 95% CI	*n* (cells)	*N* (animals)	mean ± 95% CI	*n* (cells)	*N* (animals)
Day 0	39 ± 2	114	4	36 ± 1	95	4
Day 2	41 ± 2	78	4	31 ± 1	110	4
Day 4	39 ± 8	111	4	31 ± 1	108	4
Day 6	34 ± 1	104	4	26 ± 1	102	4
I_{mean} (GAL) (a.u.)	IL-1β					
	WT			$Dcn^{-/-}$		
	mean ± 95% CI	*n* (cells)	*N* (animals)	mean ± 95% CI	*n* (cells)	*N* (animals)
Day 2	29 ± 1	131	4	27 ± 1	111	4
Day 4	23 ± 1	114	4	22 ± 1	109	4
Day 6	20 ± 1	91	4	19 ± 1	97	4

p-value (genotype/IL-1β)	WT vs $Dcn^{-/-}$		Untreated vs IL-1β	
	Control	IL-1β	WT	$Dcn^{-/-}$
Day 0	0.320	--	--	--
Day 2	0.049	0.656	0.030	0.313
Day 4	0.034	0.825	< 0.001	0.017
Day 6	0.038	0.760	0.010	0.011

p-value (time points)	Untreated		IL-1β	
	WT	$Dcn^{-/-}$	WT	$Dcn^{-/-}$
Day 0 vs 2	0.990	0.741	--	--
Day 0 vs 4	0.958	0.585	--	--
Day 0 vs 6	0.217	0.073	--	--
Day 2 vs 4	0.852	0.993	0.139	0.271
Day 2 vs 6	0.137	0.350	0.048	0.084
Day 4 vs 6	0.428	0.486	0.770	0.714

Table S2. Summary of averaged values and statistical analysis outcomes of the mean AHA intensity, I_{mean}, extracted from high-resolution confocal images of click-labeled femoral head cartilage explants of age-matched wild-type (WT) and decorin-null (*Dcn*$^{-/-}$) mice over 6 days of culture, shown as mean ± 95% CI from values averaged by each explant. The *p*-values were calculated using the linear mixed-effect model, followed by Holm-Bonferroni correction for multiple contrasts between genotypes and treatments, and Tukey-Kramer correction for multiple comparisons across culture time points for each genotype and treatment.

I_{mean} (AHA) (a.u.)	Untreated					
	WT			*Dcn*$^{-/-}$		
	mean ± 95% CI	*n* (cells)	*N* (animals)	mean ± 95% CI	*n* (cells)	*N* (animals)
Day 0	34 ± 2	76	3	35 ± 2	115	3
Day 2	34 ± 2	114	3	35 ± 2	85	3
Day 4	34 ± 2	109	3	32 ± 1	116	3
Day 6	35 ± 1	109	3	34 ± 2	99	3
I_{mean} (AHA) (a.u.)	IL-1β					
	WT			*Dcn*$^{-/-}$		
	mean ± 95% CI	*n* (cells)	*N* (animals)	mean ± 95% CI	*n* (cells)	*N* (animals)
Day 2	37 ± 2	119	3	35 ± 2	119	3
Day 4	37 ± 1	125	3	34 ± 2	113	3
Day 6	33 ± 1	123	3	38 ± 2	123	3

p-value (genotype/IL-1β)	WT vs *Dcn*$^{-/-}$		Untreated vs IL-1β	
	Untreated	IL-1β	WT	*Dcn*$^{-/-}$
Day 0	0.762	--	--	--
Day 2	1.000	0.765	1.000	0.934
Day 4	0.884	1.000	1.000	1.000
Day 6	0.968	0.945	0.596	1.000

p-value (time points)	Untreated		IL-1β	
	WT	*Dcn*$^{-/-}$	WT	*Dcn*$^{-/-}$
Day 0 vs 2	0.995	0.997	--	--
Day 0 vs 4	0.991	0.889	--	--
Day 0 vs 6	0.993	1.000	--	--
Day 2 vs 4	1.000	0.852	0.952	0.959
Day 2 vs 6	0.948	1.000	0.702	0.906
Day 4 vs 6	0.928	0.896	0.862	0.770

Table S3. Summary of averaged values and statistical analysis outcomes of the peak GAL intensity, I_{peak}, extracted from high-resolution confocal images of click-labeled femoral head cartilage explants of age-matched wild-type (WT) and decorin-null (*Dcn*$^{-/-}$) mice over 6 days of culture, shown as mean ± 95% CI from values averaged by each explant. The *p*-values were calculated using the linear mixed-effect model, followed by Holm-Bonferroni correction for multiple contrasts between genotypes and treatments, and Tukey-Kramer correction for multiple comparisons across culture time points for each genotype and treatment.

I_{peak} (GAL) (a.u.)	Untreated					
	WT			*Dcn*$^{-/-}$		
	mean ± 95% CI	*n* (cells)	*N* (animals)	mean ± 95% CI	*n* (cells)	*N* (animals)
Day 0	59 ± 2	114	4	56 ± 2	95	4
Day 2	62 ± 2	78	4	48 ± 2	110	4
Day 4	56 ± 2	111	4	46 ± 2	108	4
Day 6	49 ± 2	104	4	37 ± 2	102	4
I_{peak} (GAL) (a.u.)	IL-1β					
	WT			*Dcn*$^{-/-}$		
	mean ± 95% CI	*n* (cells)	*N* (animals)	mean ± 95% CI	*n* (cells)	*N* (animals)
Day 2	45 ± 2	131	4	45 ± 2	111	4
Day 4	33 ± 2	114	4	32 ± 2	109	4
Day 6	25 ± 1	91	4	26 ± 1	97	4

p-value (genotype/IL-1β)	WT vs *Dcn*$^{-/-}$		Untreated vs IL-1β	
	Control	IL-1β	WT	*Dcn*$^{-/-}$
Day 0	0.307	--	--	--
Day 2	0.010	0.901	0.008	0.558
Day 4	0.003	0.851	< 0.001	0.001
Day 6	0.012	0.865	0.001	0.001

p-value (time points)	Untreated		IL-1β	
	WT	*Dcn*$^{-/-}$	WT	*Dcn*$^{-/-}$
Day 0 vs 2	0.796	0.040	--	--
Day 0 vs 4	0.521	0.008	--	--
Day 0 vs 6	0.005	< 0.001	--	--
Day 2 vs 4	0.155	0.785	0.017	0.003
Day 2 vs 6	0.001	0.008	0.001	< 0.001
Day 4 vs 6	0.050	0.040	0.187	0.158

Table S4. Summary of averaged values and statistical analysis outcomes of the matrix-to-cell Euclidean distance corresponding to the peak GAL intensity, d_{peak}, extracted from high-resolution confocal images of click-labeled femoral head cartilage explants of age-matched wild-type (WT) and decorin-null ($Dcn^{-/-}$) mice over 6 days of culture, shown as mean ± 95% CI from values averaged by each explant. The p-values were calculated using the linear mixed-effect model, followed by Holm-Bonferroni correction for multiple contrasts between genotypes and treatments, and Tukey-Kramer correction for multiple comparisons across culture time points for each genotype and treatment.

d_{peak} (GAL) (μm)	Untreated					
	WT			$Dcn^{-/-}$		
	mean ± 95% CI	*n* (cells)	*N* (animals)	mean ± 95% CI	*n* (cells)	*N* (animals)
Day 0	0.91 ± 0.06	114	4	0.92 ± 0.07	95	4
Day 2	1.01 ± 0.09	78	4	1.03 ± 0.07	110	4
Day 4	1.10 ± 0.08	111	4	1.07 ± 0.08	108	4
Day 6	1.03 ± 0.07	104	4	1.05 ± 0.08	102	4
d_{peak} (GAL) (μm)	IL-1β					
	WT			$Dcn^{-/-}$		
	mean ± 95% CI	*n* (cells)	*N* (animals)	mean ± 95% CI	*n* (cells)	*N* (animals)
Day 2	0.92 ± 0.05	131	4	0.88 ± 0.07	111	4
Day 4	0.91 ± 0.08	114	4	0.90 ± 0.06	109	4
Day 6	1.00 ± 0.09	91	4	0.98 ± 0.09	97	4

p-value (genotype/IL-1β)	WT vs $Dcn^{-/-}$		Untreated vs IL-1β	
	Control	IL-1β	WT	$Dcn^{-/-}$
Day 0	0.983	--	--	--
Day 2	1.000	1.000	0.567	0.567
Day 4	1.000	1.000	0.334	0.334
Day 6	1.000	1.000	1.000	1.000

p-value (time points)	Untreated		IL-1β	
	WT	$Dcn^{-/-}$	WT	$Dcn^{-/-}$
Day 0 vs 2	0.837	0.665	--	--
Day 0 vs 4	0.491	0.496	--	--
Day 0 vs 6	0.873	0.457	--	--
Day 2 vs 4	0.927	0.991	0.974	0.989
Day 2 vs 6	1.000	0.982	0.600	0.797
Day 4 vs 6	0.897	1.000	0.482	0.717

Table S5. Summary of averaged values and statistical analysis outcomes of the Euclidean distance from d_{peak} to where the GAL intensity decays by 50%, $d_{1/2}$, extracted from high-resolution confocal images of click-labeled femoral head cartilage explants of age-matched wild-type (WT) and decorin-null ($Dcn^{-/-}$) mice over 6 days of culture, shown as mean ± 95% CI from values averaged by each explant. The *p*-values were calculated using the linear mixed-effect model, followed by Holm-Bonferroni correction for multiple contrasts between genotypes and treatments, and Tukey-Kramer correction for multiple comparisons across culture time points for each genotype and treatment.

$d_{1/2}$ (GAL) (μm)	Untreated					
	WT			$Dcn^{-/-}$		
	mean ± 95% CI	*n* (cells)	*N* (animals)	mean ± 95% CI	*n* (cells)	*N* (animals)
Day 0	0.92 ± 0.08	114	4	0.82 ± 0.10	95	4
Day 2	0.74 ± 0.11	78	4	0.78 ± 0.09	110	4
Day 4	0.74 ± 0.09	111	4	0.72 ± 0.10	108	4
Day 6	0.91 ± 0.09	104	4	0.76 ± 0.11	102	4
$d_{1/2}$ (GAL) (μm)	IL-1β					
	WT			$Dcn^{-/-}$		
	mean ± 95% CI	*n* (cells)	*N* (animals)	mean ± 95% CI	*n* (cells)	*N* (animals)
Day 2	0.90 ± 0.07	131	4	0.85 ± 0.08	111	4
Day 4	1.10 ± 0.13	114	4	0.99 ± 0.12	109	4
Day 6	0.76 ± 0.13	91	4	1.15 ± 0.17	97	4

p-value (genotype/IL-1β)	WT vs $Dcn^{-/-}$		Untreated vs IL-1β	
	Control	IL-1β	WT	$Dcn^{-/-}$
Day 0	0.762	--	--	~
Day 2	1.000	1.000	0.263	0.592
Day 4	0.887	0.486	0.087	0.087
Day 6	0.750	0.750	0.994	0.188

p-value (time points)	Untreated		IL-1β	
	WT	$Dcn^{-/-}$	WT	$Dcn^{-/-}$
Day 0 vs 2	0.698	0.976	--	--
Day 0 vs 4	0.667	0.868	--	--
Day 0 vs 6	0.996	0.988	--	--
Day 2 vs 4	1.000	0.984	0.474	0.308
Day 2 vs 6	0.820	1.000	0.922	0.032
Day 4 vs 6	0.794	0.969	0.306	0.264

Table S6. Summary of averaged values and statistical analysis outcomes of the Euclidean distance from the cell surface to where the GAL intensity decays by 50%, $d_{\text{peak}} + d_{1/2}$, extracted from high-resolution confocal images of click-labeled femoral head cartilage explants of age-matched wild-type (WT) and decorin-null (*Dcn*$^{-/-}$) mice over 6 days of culture, shown as mean ± 95% CI from values averaged by each explant. The *p*-values were calculated using the linear mixed-effect model, followed by Holm-Bonferroni correction for multiple contrasts between genotypes and treatments, and Tukey-Kramer correction for multiple comparisons across culture time points for each genotype and treatment.

$d_{\text{peak}} + d_{1/2}$ (GAL) (μm)	Untreated					
	WT			*Dcn*$^{-/-}$		
	mean ± 95% CI	*n* (cells)	*N* (animals)	mean ± 95% CI	*n* (cells)	*N* (animals)
Day 0	1.84 ± 0.09	114	4	1.73 ± 0.11	95	4
Day 2	1.75 ± 0.13	78	4	1.81 ± 0.10	110	4
Day 4	1.83 ± 0.09	111	4	1.79 ± 0.10	108	4
Day 6	1.94 ± 0.11	104	4	1.82 ± 0.13	102	4
$d_{\text{peak}} + d_{1/2}$ (GAL) (μm)	IL-1β					
	WT			*Dcn*$^{-/-}$		
	mean ± 95% CI	*n* (cells)	*N* (animals)	mean ± 95% CI	*n* (cells)	*N* (animals)
Day 2	1.82 ± 0.08	131	4	1.73 ± 0.10	111	4
Day 4	1.99 ± 0.15	114	4	1.89 ± 0.13	109	4
Day 6	1.76 ± 0.17	91	4	2.13 ± 0.18	97	4

p-value (genotype/IL-1β)	WT vs *Dcn*$^{-/-}$		Untreated vs IL-1β	
	Control	IL-1β	WT	*Dcn*$^{-/-}$
Day 0	0.726	--	--	--
Day 2	1.000	1.000	1.000	1.000
Day 4	0.799	0.799	0.369	0.369
Day 6	1.000	1.000	0.801	0.296

p-value (time points)	Untreated		IL-1β	
	WT	*Dcn*$^{-/-}$	WT	*Dcn*$^{-/-}$
Day 0 vs 2	0.983	0.965	--	--
Day 0 vs 4	0.992	0.991	--	--
Day 0 vs 6	0.902	0.853	--	--
Day 2 vs 4	0.918	0.998	0.847	0.694
Day 2 vs 6	0.742	0.986	0.951	0.121
Day 4 vs 6	0.977	0.953	0.967	0.375

Table S7. Summary of averaged values and statistical analysis outcomes of the cumulative relative percentage of GAL released from click-labeled femoral head cartilage explants of age-matched wild-type (WT) and decorin-null (*Dcn*$^{-/-}$) mice over 6 days of culture under IL-1β stimulation, shown as mean ± 95% CI from values averaged by each explant. The *p*-values were calculated using the linear mixed-effect model, followed by Holm-Bonferroni correction for multiple contrasts between genotypes and treatments, and Tukey-Kramer correction for multiple comparisons across culture time points for each genotype and treatment.

GAL release (%)	Untreated			
	WT		*Dcn*$^{-/-}$	
	mean ± 95% CI	*N*	mean ± 95% CI	*N*
Day 2	2.6 ± 0.8	6	4.5 ± 0.6	6
Day 4	4.0 ± 0.9	6	6.3 ± 0.9	6
Day 6	5.1 ± 1.0	6	7.6 ± 1.2	6
**GAL release (%)**	**IL-1β**			
	WT		*Dcn*$^{-/-}$	
	mean ± 95% CI	*N*	mean ± 95% CI	*N*
Day 2	5.7 ± 0.4	6	6.8 ± 0.7	6
Day 4	10.7 ± 0.8	6	13.0 ± 1.6	6
Day 6	14.1 ± 1.1	6	17.2 ± 1.9	6

p-value (genotype/IL-1β)	WT vs *Dcn*$^{-/-}$		Untreated vs IL-1β	
	Control	IL-1β	WT	*Dcn*$^{-/-}$
Day 2	0.041	0.248	< 0.001	0.035
Day 4	0.014	0.028	< 0.001	< 0.001
Day 6	0.009	0.009	< 0.001	< 0.001

p-value (time points)	Untreated		IL-1β	
	WT	*Dcn*$^{-/-}$	WT	*Dcn*$^{-/-}$
Day 2 vs Day 4	< 0.001	< 0.001	< 0.001	< 0.001
Day 2 vs Day 6	< 0.001	< 0.001	< 0.001	< 0.001
Day 4 vs Day 6	< 0.001	0.002	< 0.001	< 0.001

Table S8. Summary of averaged values and statistical analysis outcomes of the cumulative relative percentage of AHA released from click-labeled femoral head cartilage explants of age-matched wild-type (WT) and decorin-null (*Dcn*$^{-/-}$) mice over 6 days under IL-1β stimulation, shown as mean ± 95% confidence intervals from values averaged by each explant. The *p*-values were calculated using the linear mixed-effect model, followed by Holm-Bonferroni correction for multiple contrasts between genotypes and treatments, and Tukey-Kramer correction for multiple comparisons across culture time points for each genotype and treatment.

	Untreated			
	WT		*Dcn*$^{-/-}$	
	Mean ± 95% CI	N	Mean ± 95% CI	N
Day 2	5.1 ± 0.9	5	4.8 ± 0.5	6
Day 4	7.1 ± 0.9	5	7.1 ± 0.5	6
Day 6	8.4 ± 1.0	5	8.5 ± 0.6	6
	IL-1β			
	WT		*Dcn*$^{-/-}$	
	Mean ± 95% CI	N	Mean ± 95% CI	N
Day 2	5.4 ± 1.8	5	4.9 ± 0.7	6
Day 4	7.7 ± 1.9	5	7.7 ± 1.0	6
Day 6	9.2 ± 2.2	5	9.4 ± 1.1	6

p-value (genotype/IL-1β)	WT vs *Dcn*$^{-/-}$		Untreated vs IL-1β	
	Control	IL-1β	WT	*Dcn*$^{-/-}$
Day 2	1.000	1.000	1.000	1.000
Day 4	1.000	1.000	0.679	0.679
Day 6	1.000	1.000	0.545	0.332

p-value (time points)	Untreated		IL-1β	
	WT	*Dcn*$^{-/-}$	WT	*Dcn*$^{-/-}$
Day 2 vs Day 4	< 0.001	< 0.001	< 0.001	< 0.001
Day 2 vs Day 6	< 0.001	< 0.001	< 0.001	< 0.001
Day 4 vs Day 6	0.007	< 0.001	< 0.001	< 0.001

Table S9. Summary of averaged values and statistical analysis outcomes of the cumulative relative percentage of GAL released from click-labeled femoral head cartilage explants of age-matched wild-type (WT) and decorin knockout (*Dcn*$^{-/-}$) mice over 8 days of culture under IL-1β stimulation with the filtration of exogenous decorin either before or after the onset of IL-1β (Pre-Dcn, Post-Dcn, Comb-Dcn), shown as mean ± 95% CI from values averaged by each explant. The *p*-values were calculated using the linear mixed-effect model, followed by Holm-Bonferroni correction for multiple contrasts between genotypes, and Tukey-Kramer correction for multiple comparisons among different treatment conditions for each genotype and time point, and across culture time points for each genotype and treatment.

	WT									
	Untreated		IL-1β only		Pre-Dcn		Post-Dcn		Comb-Dcn	
	mean ± 95% CI	*N*	mean ± 95% CI	*N*	mean ± 95% CI	*N*	mean ± 95% CI	*N*	mean ± 95% CI	*N*
Day 2	2.8 ± 0.6	7	8.1 ± 1.2	6	7.9 ± 1.2	10	7.9 ± 1.3	10	7.7 ± 1.1	5
Day 4	5.1 ± 0.4	7	12.6 ± 0.7	6	12.2 ± 1.4	10	14.6 ± 1.3	10	13.2 ± 1.1	5
Day 6	6.6 ± 0.3	7	16.6 ± 0.9	6	14.5 ± 1.2	10	19.2 ± 1.4	10	15.6 ± 1.1	5
Day 8	8.1 ± 0.3	7	24.5 ± 2.2	6	16.4 ± 1.3	10	26.9 ± 2.3	10	17.6 ± 1.1	5
	Dcn$^{-/-}$									
	Untreated		IL-1β only		Pre-Dcn		Post-Dcn		Comb-Dcn	
	mean ± 95% CI	*N*	mean ± 95% CI	*N*	mean ± 95% CI	*N*	mean ± 95% CI	*N*	mean ± 95% CI	*N*
Day 2	3.9 ± 0.5	6	10.8 ± 1.8	7	9.4 ± 1.1	11	10.6 ± 0.9	8	9.8 ± 1.8	4
Day 4	7.9 ± 1.1	6	15.7 ± 0.9	7	15.0 ± 1.3	11	16.4 ± 1.0	8	14.9 ± 1.8	4
Day 6	9.7 ± 1.3	6	19.7 ± 1.8	7	20.3 ± 2.7	11	19.7 ± 1.3	8	18.5 ± 1.8	4
Day 8	11.2 ± 1.7	6	28.4 ± 1.2	7	29.7 ± 1.3	11	31.2 ± 1.5	8	32.0 ± 2.4	4

p-value (treatment conditions)		WT				*Dcn*$^{-/-}$			
		IL-1β only	Pre-Dcn	Post-Dcn	Comb-Dcn	IL-1β only	Pre-Dcn	Post-Dcn	Comb-Dcn
Day 2	Untreated	< 0.001	< 0.001	< 0.001	< 0.001	< 0.001	< 0.001	< 0.001	0.002
	IL-1β only	--	1.000	1.000	0.998	--	1.000	0.720	0.946
	Pre-Dcn	--	--	1	1.000	--	--	0.808	0.974
	Post-Dcn	--	--	--	1.000	--	--	--	0.999
Day 4	Untreated	< 0.001	< 0.001	< 0.001	< 0.001	< 0.001	< 0.001	< 0.001	< 0.001
	IL-1β only	--	0.993	0.338	0.998	--	0.976	0.971	0.984
	Pre-Dcn	--	--	0.071	0.884	--	--	0.688	0.836
	Post-Dcn	--	--	--	0.735	--	--	--	1
Day 6	Untreated	< 0.001	< 0.001	< 0.001	< 0.001	< 0.001	< 0.001	< 0.001	< 0.001
	IL-1β only	--	0.303	0.106	0.943	--	1	0.983	0.935
	Pre-Dcn	--	--	< 0.001	0.856	--	--	0.982	0.925
	Post-Dcn	--	--	--	0.018	--	--	--	0.699
Day 8	Untreated	< 0.001	< 0.001	< 0.001	< 0.001	< 0.001	< 0.001	< 0.001	< 0.001
	IL-1β only	--	< 0.001	0.147	< 0.001	--	0.1491	0.765	0.111
	Pre-Dcn	--	--	< 0.001	0.790	--	--	0.652	0.981
	Post-Dcn	--	--	--	< 0.001	--	--	--	0.460

Table S9. (Continued)

p-value (genotypes)	WT vs *Dcn*$^{-/-}$				
	Untreated	IL-1β only	Pre-Dcn	Post-Dcn	Comb-Dcn
Day 2	0.312	0.072	0.047	0.312	0.312
Day 4	0.002	0.029	0.002	0.774	0.415
Day 6	< 0.001	0.024	< 0.001	0.366	0.080
Day 8	< 0.001	0.002	< 0.001	0.030	< 0.001

p-value (time points)	WT				
	Untreated	IL-1β only	Pre-Dcn	Post-Dcn	Comb-Dcn
Day 2 vs 4	< 0.001	0.002	< 0.001	< 0.001	< 0.001
Day 2 vs 6	< 0.001	< 0.001	< 0.001	< 0.001	< 0.001
Day 2 vs 8	< 0.001	< 0.001	< 0.001	< 0.001	< 0.001
Day 4 vs 6	< 0.001	0.005	< 0.001	0.003	< 0.001
Day 4 vs 8	< 0.001	< 0.001	< 0.001	< 0.001	< 0.001
Day 6 vs 8	< 0.001	< 0.001	< 0.001	< 0.001	0.004
p-value (time points)	*Dcn*$^{-/-}$				
	Untreated	IL-1β only	Pre-Dcn	Post-Dcn	Comb-Dcn
Day 2 vs 4	< 0.001	0.002	< 0.001	< 0.001	< 0.001
Day 2 vs 6	< 0.001	< 0.001	< 0.001	< 0.001	< 0.001
Day 2 vs 8	< 0.001	< 0.001	< 0.001	< 0.001	< 0.001
Day 4 vs 6	0.065	0.007	< 0.001	< 0.001	0.006
Day 4 vs 8	0.001	< 0.001	< 0.001	< 0.001	< 0.001
Day 6 vs 8	0.148	< 0.001	< 0.001	< 0.001	< 0.001

Table S10.: Summary of fitting outcomes for the ternary bindings measured by surface plasmon resonance (SPR), including the apparent association and dissociation rate constants (k_a and k_d), as well as the equilibrium dissociation constant (K_D), shown for values of each individual replicate calculated from Scrubber, as well as mean ± SD for the fold changes of $1/K_D$ measured on each individual experiment with versus without decorin as the coupler repeated for n = 3 replicates.

Ligand	Analyte	Without Decorin			With Decorin			Fold change in $1/K_D$	mean ± SD of fold change in $1/K_D$
		k_a ($\times 10^4$ $M^{-1}\cdot s^{-1}$)	k_d ($\times 10^{-3}$ s^{-1})	K_D (nM)	k_a ($\times 10^4$ $M^{-1}\cdot s^{-1}$)	k_d ($\times 10^{-3}$ s^{-1})	K_D (nM)		
Aggrecan	Aggrecan	27 ± 2	(9.5 ± 0.1) × 10^2	(3.5 ± 0.3) × 10^3	67 ± 3	(1.1 ± 0.1) × 10^3	(1.6 ± 0.1) × 10^3	2.20	2.7 ± 0.8
		(8.8 ± 0.5) × 10^2	(6.0 ± 0.1) × 10^2	68 ± 1	(2.1 ± 0.1) × 10^3	(6.7 ± 0.1) × 10^2	31 ± 1	2.19	
		(2.9 ± 0.1) × 10^2	(4.4 ± 0.1) × 10^2	(1.5 ± 0.1) × 10^2	(7.5 ± 0.6) × 10^2	(3.1 ± 0.1) × 10^2	42 ± 2	3.62	
Collagen	Aggrecan	4.1 ± 0.1	15.4 ± 0.2	(3.7 ± 0.1) × 10^2	5.0 ± 0.1	7.9 ± 0.1	157 ± 4	2.36	2.3 ± 0.1
		(1.7 ± 0.1) × 10^3	(6.4 ± 0.1) × 10^2	36 ± 1	(4.4 ± 0.1) × 10^3	(6.5 ± 0.1) × 10^2	14.7 ± 0.2	2.46	
		8.9 ± 0.1	2.7 ± 0.1	30 ± 1	10.0 ± 0.1	1.38 ± 0.03	13.7 ± 0.2	2.20	
Aggrecan	Collagen	8.2 ± 0.3	(5.3 ± 0.1) × 10^2	(6.5 ± 0.2) × 10^3	23 ± 1	(4.1 ± 0.1) × 10^2	(1.8 ± 0.1) × 10^3	3.57	3.4 ± 1.6
		15.1 ± 0.8	(6.1 ± 0.1) × 10^2	(4.0 ± 0.2) × 10^3	(1.1 ± 0.1) × 10^2	(8.6 ± 0.1) × 10^2	(8.2 ± 0.2) × 10^2	4.88	
		1.4 ± 0.2	(6.3 ± 0.1) × 10^2	(4.6 ± 0.6) × 10^4	2.2 ± 0.2	(6.1 ± 0.1) × 10^2	(2.7 ± 0.1) × 10^4	1.70	
Collagen	Collagen	24 ± 1	0.8 ± 0.1	3.4 ± 0.5	30 ± 1	0.43 ± 0.01	1.4 ± 0.1	2.33	1.7 ± 0.6
		27 ± 1	4.6 ± 0.1	17 ± 1	26 ± 4	3.5 ± 0.1	13.5 ± 0.2	1.27	
		35 ± 1	8.3 ± 0.1	24 ± 1	29 ± 1	4.4 ± 0.1	15.1 ± 0.2	1.58	

Table S11. Summary of averaged values and statistical analysis outcomes of the indentation modulus, E_{ind}, of femoral condylar cartilage from age-matched wild-type (WT) and decorin-null (*Dcn*$^{-/-}$) mice subjected to enzymatic GAG-depletion and re-infiltration with exogenous decorin, shown as mean ± 95% CI from values averaged by animal. The *p*-values were calculated using the linear mixed-effect model, followed by Holm-Bonferroni correction for multiple contrasts between genotypes and IL-1β stimulations, and Tukey-Kramer correction for multiple comparisons across different digestion and decorin infiltration conditions.

E_{ind} (kPa)		− IL-1β				+ IL-1β		
		Undigested	Undigested + Dcn	GAG-dep.	GAG-dep. + Dcn	Undigested	GAG-dep.	GAG-dep. + Dcn
WT	mean ± 95% CI	397 ± 81	310 ± 77	122 ± 17	253 ± 65	160 ± 21	152 ± 50	133 ± 16
	N	12	8	17	8	9	5	5
Dcn$^{-/-}$	mean ± 95% CI	159 ± 37	153 ± 20	142 ± 33	149 ± 30	158 ± 37	155 ± 43	150 ± 31
	N	12	6	11	14	7	8	9
p-value (genotype)		< 0.001	0.009	0.108	0.012	1.000	1.000	1.000

p-value (treatments)	WT			
	Undigested vs GAG-dep.	Undigested vs Undigested + Dcn	Undigested vs GAG-dep. + Dcn	GAG-dep. vs GAG-dep. + Dcn
− IL-1β	< 0.001	0.524	0.019	0.015
+ IL-1β	0.897	--	0.971	0.991
p-value (treatments)	*Dcn*$^{-/-}$			
	Undigested vs GAG-dep.	Undigested vs Undigested + Dcn	Undigested vs GAG-dep. + Dcn	GAG-dep. vs GAG-dep. + Dcn
− IL-1β	0.751	0.751	0.751	0.751
+ IL-1β	0.996	--	0.960	0.981

p-value (IL-1β)	Undigested	GAG-dep.	GAG-dep. + Dcn
WT	< 0.001	0.084	0.048
Dcn$^{-/-}$	1.000	1.000	1.000

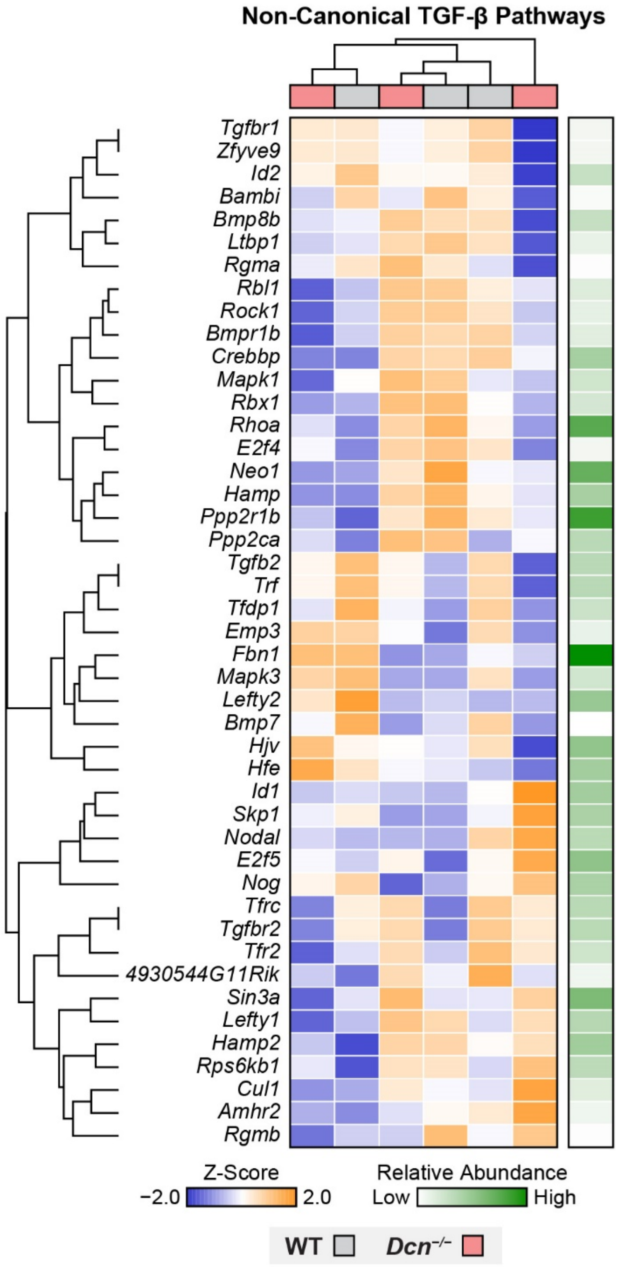

Figure S1. Heatmap of unbiased clustering for genes involved in KEGG non-canonical TGF-β signaling pathways show no clear separation in signaling activities between 3-month-old WT and *Dcn*$^{-/-}$ cartilage (n = 3 per genotype).

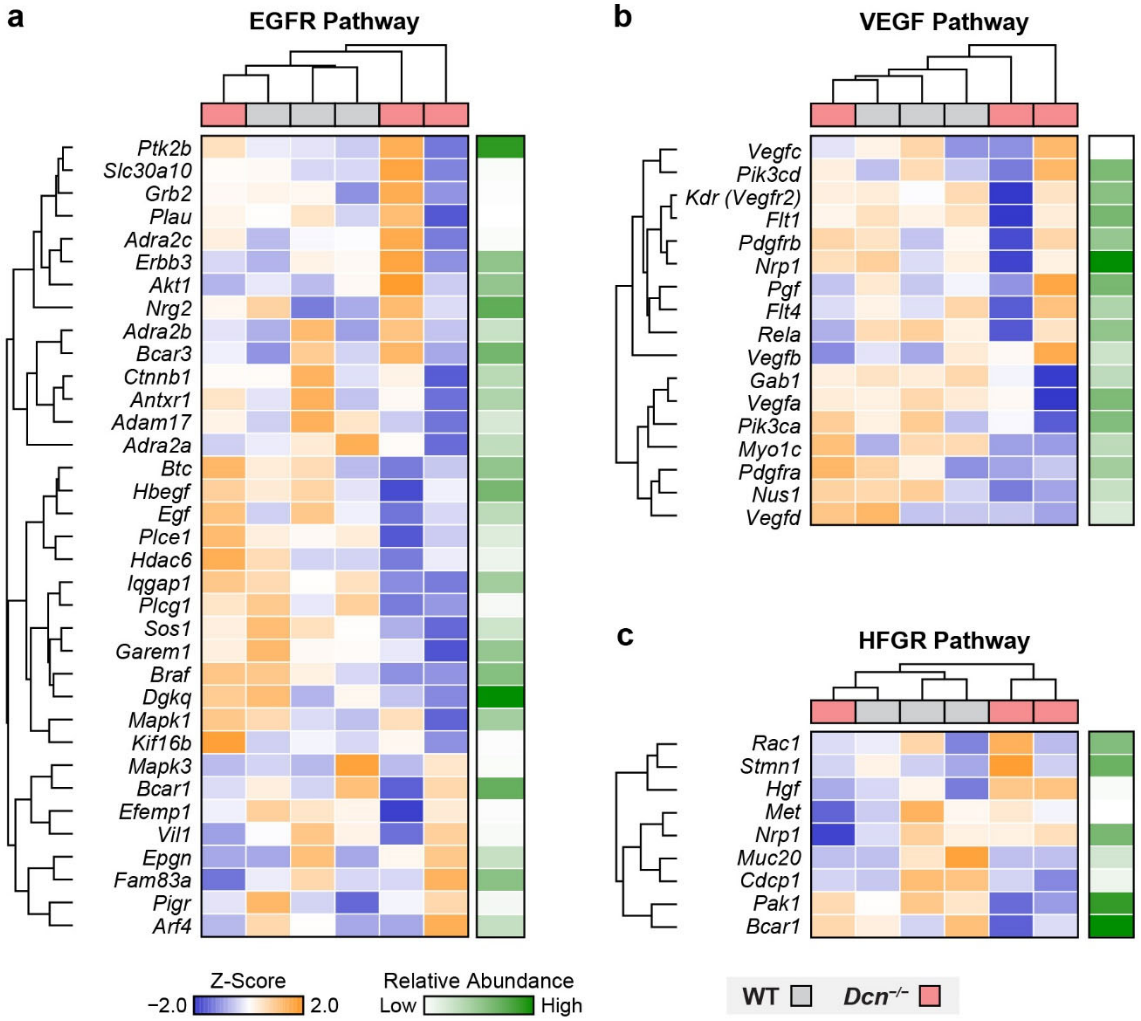

Figure S2. Heatmap of unbiased clustering for genes involved in other pathways potentially mediated by decorin from the GO database show no clear separation in these signaling activities between 3-month-old WT and *Dcn*$^{-/-}$ cartilage (*n* = 3 per genotype): a) EGFR pathway, b) VEGF pathway, c) HFGR pathway.

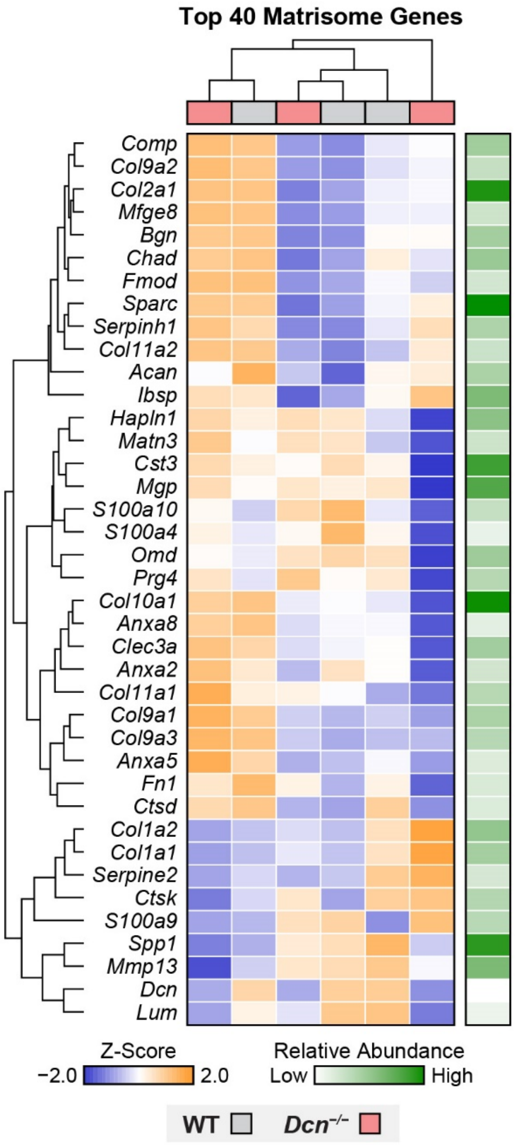

Figure S3. Heatmap of unbiased clustering of top 40 genes from the GO Matrisome Database reveals no clear separation in matrix biosynthesis activities between WT and *Dcn*$^{-/-}$ cartilage (n = 3 genotype).

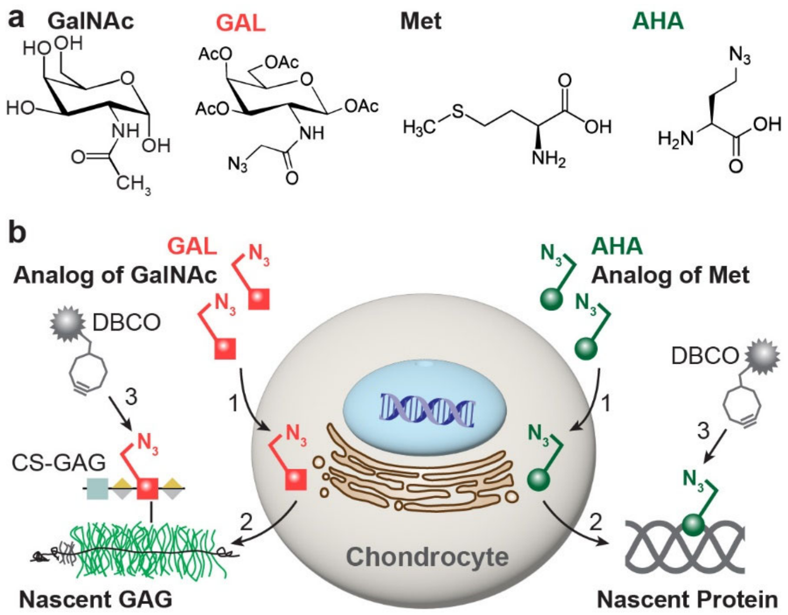

Figure S4. Schematic illustration of click-labeling for nascent glycosaminoglycans (GAGs) and proteins. a) Molecular structures of *N*-acetylgalactosamine (GalNAc), *N*-azidoacetylgalactosaminetetraacylated (GAL), methionine (Met), and azidohomoalanine (AHA). b) The azide-modified monosaccharide GAL serves as an analog of GalNAc and is metabolically incorporated into newly synthesized GAGs. Similarly, azidohomoalanine AHA, an analog of methionine, is incorporated into newly synthesized proteins. The click-labeling reagent, e.g., dibenzocyclooctyne (DBCO), is then applied to label GAL-containing nascent GAGs and AHA-containing nascent proteins via bio-orthogonal copper-free click chemistry, enabling their visualization and quantitative analysis.

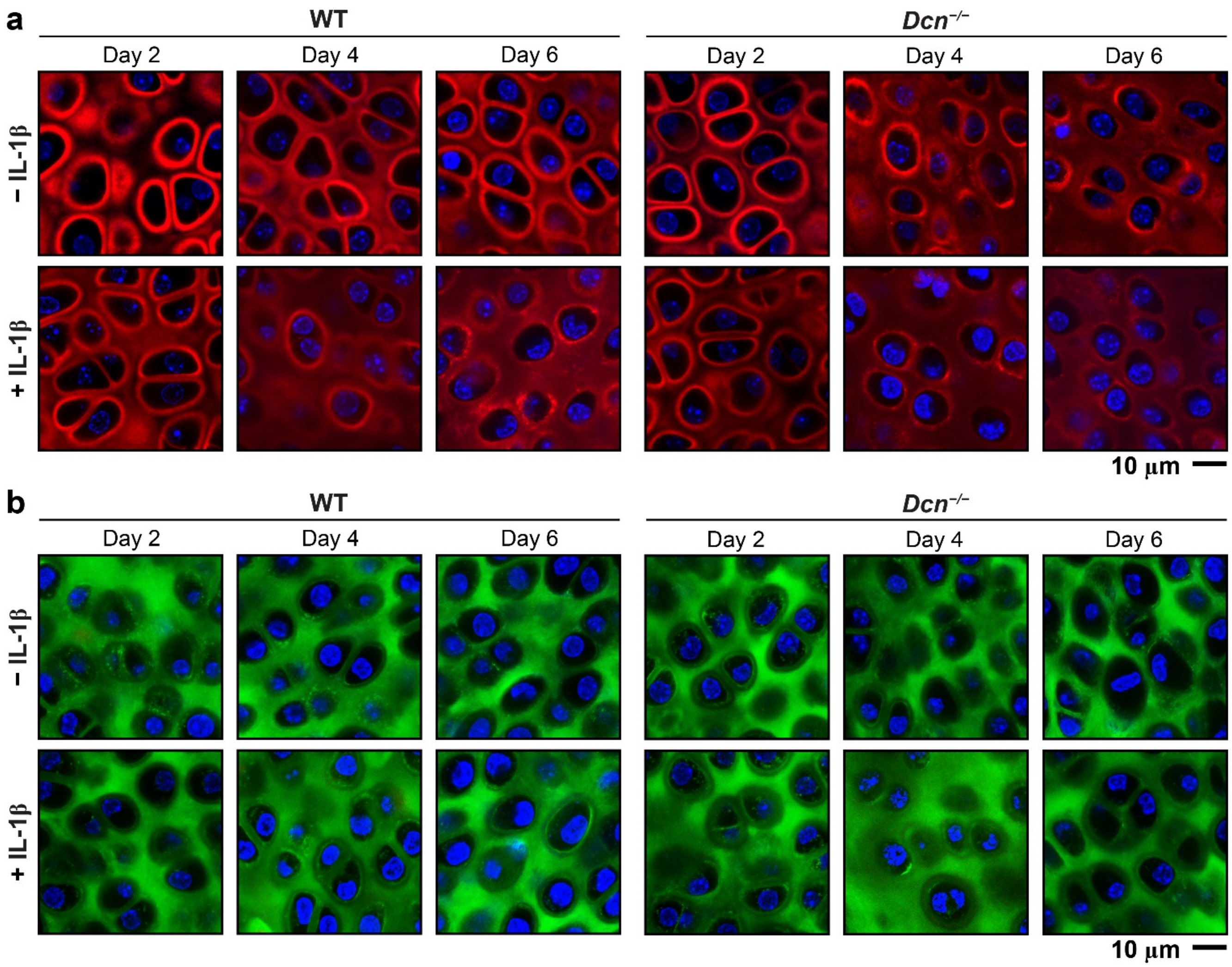

Figure S5. Representative confocal images of GAL (red) and AHA (green) labeling in 3-week-old wild-type (WT) and decorin-null (*Dcn*$^{-/-}$) cartilage explants cultured for 2, 4 and 6 days with or without IL-1β stimulation. Results show preferential localization of nascent GAGs in the pericellular matrix (PCM) and widespread distribution of nascent proteins throughout the extracellular matrix (ECM).

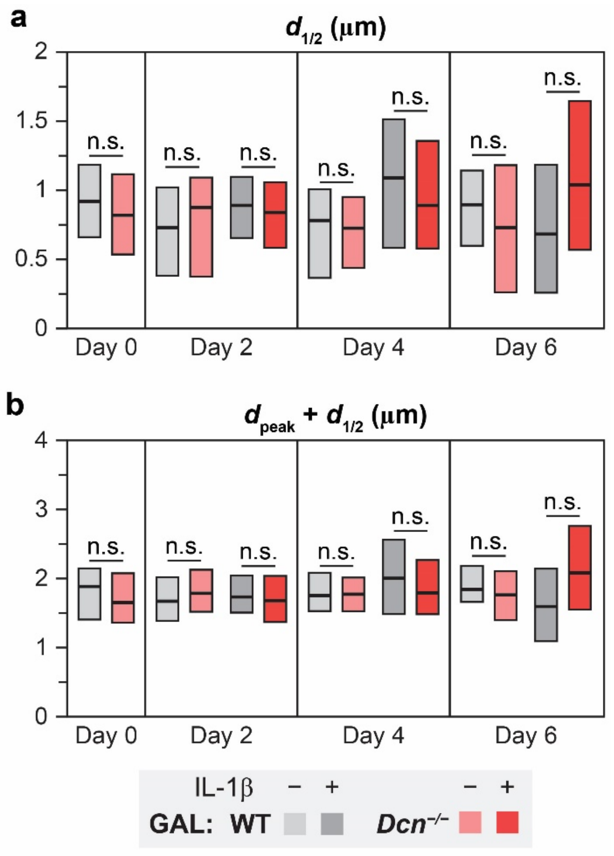

Figure S6. Loss of decorin does not affect additional spatial parameters of GAL signals between wild-type (WT) and decorin-null (*Dcn*$^{-/-}$) cartilage from day 0 to day 6 of culture with or without IL-1β: a) $d_{1/2}$ and b) $d_{peak} + d_{1/2}$. Box plots represent data obtained from > 75 ROIs across $n \geq 4$ animals for each group, n.s.: not significant.

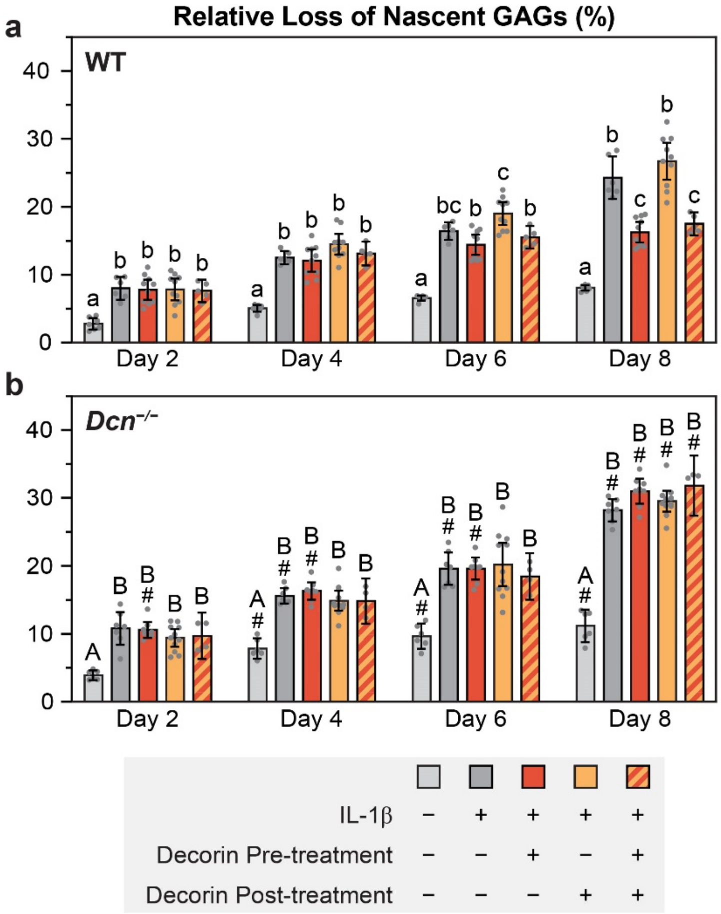

Figure S7. Cumulative release of nascent GAGs from a) WT and b) *Dcn*$^{-/-}$ cartilage explants at days 2, 4, 6 and 8 of culture under various decorin treatment conditions with IL-1β stimulation. Each data point represents one biological replicate (mean ± 95% CI, $n \geq 4$). Different letters indicate significant differences among treatments within each genotype and time point. $^{\#}$: $p < 0.05$ between genotypes from the same treatment and time point.
